# Surgical Decisions in Primary Open Angle Glaucoma with Low or Normal Tension

**DOI:** 10.5005/jp-journals-10008-1149

**Published:** 2013-09-06

**Authors:** JW Shum, Dy Leung

**Affiliations:** Department of Ophthalmology, The Eye Institute, Queen Mary Hospital, University of Hong Kong, Hong Kong; Department of Ophthalmology, Hong Kong Sanatorium and Hospital; Department of Ophthalmology and Visual Sciences Hong Kong Eye Hospital, The Chinese University of Hong Kong, Hong Kong

**Keywords:** Normal tension glaucoma, Glaucoma surgery, Low tension glaucoma, IOP control, Deep sclerectomy, Trabeculectomy.

## Abstract

Glaucoma, given its insidious nature, is often coined ‘the sneak thief of sight'. Following this trail of thought, primary open angle glaucoma with low or normal tension (POAGLNT) could be coined ‘the king of thieves'. The lack of a compelling red fag of high intraocular pressure (IOP), together with the diurnal fuctuation of the deceptively low baseline IOP, POAGLNT poses a therapeutic challenge in terms of judging when to intervene, and how.

In this review article, we will outline the considerations before undergoing surgery: risk stratification, defining goal in terms of target pressure and IOP modulation. We will also review the strengths, weaknesses and pearls of available options.

**How to cite this article:** Shum JW, Leung DY. Surgical Decisions in Primary Open Angle Glaucoma with Low or Normal Tension. J Current Glau Prac 2013;7(3):121-127.

## INTRODUCTION

Primary open-angle glaucoma (POAG) with high tension, and Primary open-angle glaucoma with low or normal tension (POAGLNT), or normal-tension glaucoma (NTG) are not separate entities and represent a continuum. Where intraocular pressure (IOP) plays a predominant role in POAG, potentially more vascular and other risk factors come into play, interacting with IOP across its entire range, in the pathogenesis of POAGLNT.

POAGLNT has a high prevalence among OAG, data suggested a prevalence of 60-80% among Asians and Blacks, up to 92% in Japan, and at least 33% or more for Caucasians populations.^1-7^

### Target Pressure in NTG

The term target pressure was first coined by Dr Paul Palmberg at Bascom Palmer Eye Institute in the 1950s. It was first recommended by the American Academy of Ophthalmology in the 1990s as part of the preferred practice pattern for POAG. It signifes the pressure at which clinicians expects that the rate of ganglion cell loss to be no greater than the age-dependent rate or the highest IOP at which no more clinically apparent glaucomatous optic nerve damage occurs.^8^

Different working definitions have arisen. In management of POAGLNT or NTG, collaborative normal tension glaucoma study (CNTGS) adopted a percentage approach, and found that IOP lowering of 30% from baseline was effective in slowing disease progression.^9^ Alternatively some Japanese and Korean studies suggests a numerical target of lowering of IOP ≤ 10 mm Hg from baseline.^10^ More studies will be needed before we know whether a universal target pressure is applicable in POAGLNT, or whether different target pressures may be needed for different subtypes of POAGLNT.

The role of presenting IOP in POAGLNT was studied in Hong Kong by comparing the rate of progression and clinical characteristics.^14^ Subjects were defined as high-teens NTG (HNTG) or low-teens NTG (LNTG) depending on whether their maximal untreated office IOP was greater or lesser than 15 mm Hg respectively. Four hundred and thirty subjects were followed for 3 years, and ¼ of subjects fell into LNTG category with a mean untreated IOP of around 12 mm Hg. LNTG was found to be significantly associated with advanced age, thinner CCT, systemic hypertension and renal failure. Progression rate and the incidence of disk hemorrhage were comparable between HNTG and LNTG. Careful contemplation of the risk-benefit ratio of any aggressive treatment in LNTG is advised, taking their projected lifespan, quality of life and disease progression into account. More studies are needed to better define the best treatment strategy for LNTG.

For POAGLNT, the target pressure is no easy goal to achieve with medications alone. Studies comparing the achievability of 30% IOP lowering using medications and surgery found about approximately 50% success rate using topical medication and/or laser trabeculoplasty, with the remaining patients requiring fltering surgery.^11^ One study reviewed the success rate of trabeculectomy with mitomycin-C (MMC) in achieving this target IOP, and found it to be around 40% 4 years after surgery.^12^

The possible narrower safety margin and complication rates associated with traditional glaucoma surgery in POAGLNT requires attention. Some evidence suggested that hypotony and its associated complications may be more likely to occur in fltration procedures like trabeculectomy in POAGLNT.^51,52^ As we shall detail below, options including nonpenetrating trabecular surgery may offer a safer route. In the long run, researchers need to probe deeper into analysing the cost-effectiveness associated with various treatment modalities in POAGLNT.

In CNTGS, the beneficial effect of trabeculectomy in terms of visual field (VF) outcome was only statistically significant after correcting the data for cataract development. A systemic review of the Cochrane Database reviewed randomized controlled trials comparing various medical and surgical interventions of NTG (with only 8 studies out of 398 articles meeting this criteria) in 2003, and had also concurred with the above view.^13^ The evidence raises issues on whether targeting IOP through medical or surgical means would translate into clinically relevant preservation of visual field, vision and overall gain in health-related quality of life (HRQOL).^8^ Further prospective randomized studies are required to further shed light on this issue.

From CNTGS data it is therefore recommendable that we should remove the cataract in NTG patients whenever feasible, otherwise the treatment benefit of IOP reduction will be masked.

### Risk Stratification

In practice, most clinicians adopt the approach of lowering the IOP based on the risk of progression and amount of glaucomatous damage already present. Numerous risk factors have been identified to be associated with VF progression (Table 1).

As target pressure can be difficult to safely achieve in POAGLNT, a glaucomatologist may like to assess the patient's risk of progression, before discussing with the patient the risks and benefits of achieving the target pressure via surgical means.

The abundance of risk factors one has to consider gives rise to difficulty in determining the individual risk of a patient, as sometimes it is difficult to appreciate the relative magnitude of effects of various risk factors upon a same individual. Researchers have attempted to surmount this problem using mathematical models to predict the risk of progression. Pressure-to-cornea index (PCI) is a unifed index taking into consideration the impact of two independent glaucoma risk factors – IOP and central cornea thickness (CCT).^15^ Based on this concept, we worked on Pressure-cornea-vascular index (PCVI), an adaptation of PCI specifically for POAGLNT, which extends the equation by including risk factors, especially vascular ones, identifed as associated with VF progression in a prospective POAGLNT cohort.^16^ Four hundred and fifteen eyes from 415 subjects with 3 years of follow-up were analyzed. Among the subjects, 184 showed field progression while the remaining 231 were stable. The construct of PCVI is as follows: [Max pretreatment office IOP × age at presentation × vertical CDR at presentation × (1.5 if presence of systemic hypertension; 2.5 if presence of disk hemorrhage; 3.5 if presence of both; 1.0 if none)]/[(CCT^3)*100] (CCT in mm). This gave the highest area under curve at 0.71 (95% CI = 0.66 to 0.76, p < 0.001), which is comparable to established scoring systems in neurovascular medicine (such as Framingham's scoring for stroke). The mean PCVI were 113.1 ± 76.8 and 69.7 ± 39.7 for progressed and stable POAGLNT groups respectively (p < 0.001). Thus, PCVI can be used to predict the risk of progression and is useful in individualizing treatment/surgical decisions.

Moreover, the importance of fundamental basics cannot be overemphasized. Proper documentation of progression, the rate of progression, glaucoma medication compliance check and enhancement many a times helps avoid surgery.

**Table 1:** **Table 1:** Some of the risk factors for VF progression in major eye studies

*Factors*		*Studies*	
Increasing age		AGIS, CIGTS, EMGT, OHTS, EGPS	
African ancestry		AGIS, CIGTS, CNTGS	
VF severity		EMGTS, AGIS, OHTS, EGPS	
Diabetes mellitus		AGIS, CIGTS	
Disk hemorrhage		EMGT, CNTGS	
Follow-up IOP		EMGT, CNTGS	
Cup-to-disk ratio		OHTS, EGPS	
Central corneal thickness		OHTS, EGPS	
Pseudoexfoliation syndrome		EMGT	
Presentation IOP		EMGT	
Female gender		CNTGS	
Male gender		AGIS	

*Note*: AGIS: Advanced glaucoma intervention study; CIGTS: Collaborative initial glaucoma treatment study; EMGT: Early manifest glaucoma trial; OHTS: Ocular hypertension treatment study; EGPS: European glaucoma prevention study; CNTGS: Collaborative normal tension glaucoma study

### IOP Fluctuation and Modulation

Nouri-Mahdavi et al explored the predictive factors for VF progression in advanced glaucoma intervention study (AGIS), and long-term IOP fuctuation remained a strong predictor of VF deterioration despite inclusion of mean IOP and number of surgical interventions as independent variables in the models.^17^ Some trials showed discrepant findings with regard to the detrimental effect of IOP fuctuation.^18^ However, a number of studies have consistently shown greater IOP fuctuation to be damaging in eyes with low mean IOP.^19-21^

One fact we do have consensus on is the fact that IOP fuctuation remains a tricky parameter to measure. The actual IOP fuctuation is infuenced by a multitude of factors: diurnal rhythm, perfusion pressure, translaminar pressure gradient, the cardiac cycle, blinking and eye rubbing. Should we measure short-term IOP fluctuation within 24 hours or long-term IOP fuctuation on separate days? Should it be represented by peak, range or standard deviation? Technological advancement in diurnal IOP measurement and their incorporation into future studies would further delineate the association between IOP fuctuation parameters and field progression.

### Choices, Choices…

### Trabeculectomy *vs* Deep Sclerectomy

Trabeculectomy, with its long-established history, remains widely practiced for glaucoma. Studies are robust in supporting its efficacy in lowering IOP and even reducing field progression in normal tension glaucoma.^22-24^

The literature has conflicting views regarding its efficacy in IOP modulation. Medeiros et al compared the diurnal tension curve, peak IOP and response to water-drinking test (WDT) between patients that have undergone trabeculectomy and those that are on ocular hypotensive therapy. They found that the surgical group had significantly less fuctuation of the diurnal tension curve, peak IOP and also following WDT.^25^ Ross et al again compared similar arms, and found reduction in mean IOP, peak IOP but no reduction in diurnal fuctuation.^26^ Hirooka et al found a reduction in mean IOP, and a significant reduction in IOP fuctuation with pressure change post-trabeculectomy.^27^

However, trabeculectomy is well known to predispose patients to complications such as bleb-related complications, hyphema, fat anterior chamber, long lasting hypotony, infammation, and an increased rate of cataract formation.^28,29^ In CNTGS, the incidence of cataract formation in patients who under trabeculectomy were 2 to 3 times higher than those on medications.^30^ In collaborative initial glaucoma treatment study (CIGTS) found similar results after taking the aging factor into account.^31^

One study has looked into the choice of antimetabolites and found that the use of MMC is associated with a greater risk of VF progression despite a greater fall in IOP, which may be related to the higher rate of late postoperative complications when compared with the use of 5-FU.^32^ One must respect the fine balance between targeting a low IOP and the narrow range of safety margin.

While there is sufficient data nowadays to demonstrate the efficacy of nonpenetrating deep sclerectomy (NPDS) in POAG, its effect on POAGLNT remains less well studied. Suominen et al followed the IOP reduction in POAGLNT patients after deep sclerectomy with a collagen implant and MMC.^33^ One year results were satisfactory with mean IOP reduction of 37% from baseline in 21 eyes. Ten eyes required laser goniopuncture to achieve this IOP, but no complications related to postoperative hypotony were encountered.

**Fig. 1:**
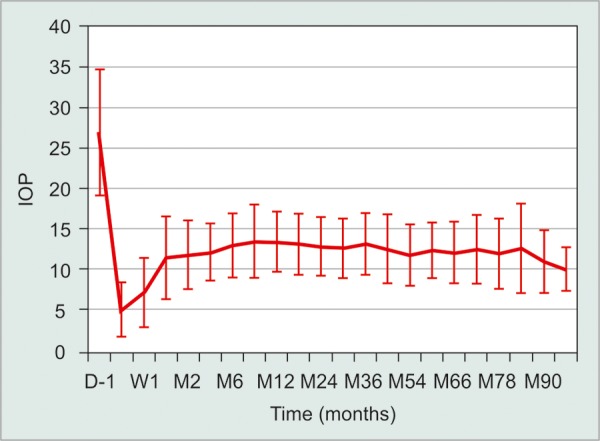
Intraocular pressures (IOP) before and after deep sclerectomy with collagen implant. (Adapted from Shaarawy T et al, 2004)

Long-term IOP control also appears reasonable in deep sclerectomy with collagen implants, according to Shaarawy et al in a prospective trial of 105 eyes^34^ (Fig. 1). Again, no postoperative hypotony or bleb-related complications were encountered, and around 50% of patients required goniopuncture. Studies comparing NPDS and trabeculectomy in OAG showed that IOP in NPDS to be comparable or slightly lower than trabeculectomy, but consistently showed less complications.^35-38^

For IOP modulation, a recent study compared the diurnal fuctuation and response with water drinking test (WDT) between patients on latanoprost, trabeculectomy and deep sclerectomy with collagen implant (DSCI).^39^ The diurnal variation was comparable between the 3 groups. With the WDT, the IOP fuctuation was significantly higher among patients treated with latanoprost when compared to those who underwent trabeculectomy and DSCI.

In summary, trabeculectomy may be a better method when a large reduction IOP is the primary goal. However, we must bear in mind the higher complication and cataract progression rate. NPDS can offer moderate IOP reduction comparable to trabeculectomy, can also achieve IOP modulation, and is safer, rendering ambulatory care easier.

### Authors' Comments

In trabeculectomy for POAGLNT, I explain to my patients sometimes we need initial mild to moderate hypotony to allow better bleb survival and target pressure achievement. I am in favor of the use of moderate MMC dosage (not excessive, e.g. 0.2 mg/ml for 1 minute), and meticulous use of laser suture lysis and adjustable sutures to help me attain this. Very judicious use of mild amount of viscoelastics in anterior chamber help to tie over the initial hypotony period and can help to maintain anterior chamber stability.

Trabeculectomy or tubes tend to give a lower target pressure. The main benefit of NPDS is that it is very safe, despite in the long run, in my experience with the Chinese subjects, more than half will require YAG laser goniopuncture for enhancement. NPDS together with collagen implant in a same operation with Phacoemulsification and intraocular lens implantation has become one of my preferred option for NTG requiring glaucoma surgery.

### Phacocanaloplasty

Canaloplasty entails dilatation of the canal of Schlemm with viscoelastic followed by placement of a microcatheter, effectively enhancing drainage without the creation of a bleb. This is considered by many to be a feasible option for patients at high risk for the visual-threatening complications of trabeculectomy.

**Fig. 2:**
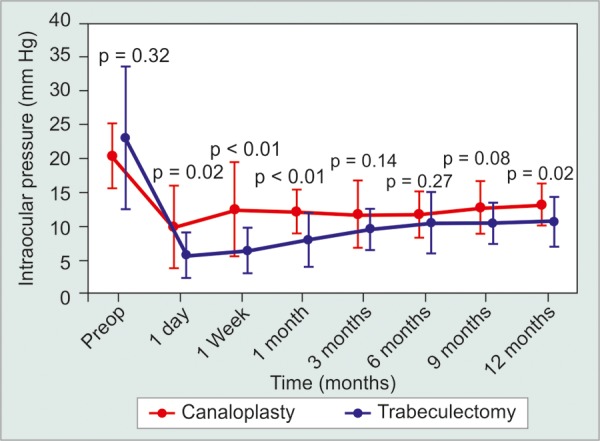
Comparison of mean intraocular pressure (IOP) between canaloplasty and trabeculectomy groups after excluding surgical failures and patients taking postoperative medications at 12 months. Significant mean IOP differences are shown in bold over a 12-month period (Adapted from Ayyala RS et al, 2011)

Study results comparing its surgical outcome to traditional methods are emerging. Decrease in IOP, failure rate and complication rates for 33 eyes undergoing canaloplasty and 46 eyes undergoing trabeculectomy at 1 year was compared.^40^ The mean reduction from baseline IOP was 32% for the canaloplasty group and 43% for the trabeculectomy group (Fig. 2). Failure rate for the two groups was 12 and 4% for the two groups respectively. Note that the above figures are not statistically significant.

The most common complication in the canaloplasty group was hyphema (21%). Hyphema has been thought to be a positive prognostic indicator signifying good aqueous outflow. There were two cases of peripheral anterior synechiae and 1 case of Descemet's membrane detachment. Descemet's membrane detachment may occasionally occur with intracorneal hemorrhage. This can be managed conservatively, or with needle aspiration, washout, gas tamponade or YAG laser descemet's membrane puncture, although there has been report of corneal decompensation requiring keratoplasty.^41^

Another study comparing the success rate of achieving 30% IOP reduction from baseline between patients undergoing phacotrabeculectomy and phacocanaloplasty revealed 79 and 60% success rate at 1 year respectively.^42^

Combination of canaloplasty with phacoemulsification have been shown to result in decreased number of glaucoma medications and an extended duration of the IOP lowering effect of phacoemulsification alone.^43^

Overall, the profle of complications for canaloplasty seems to be safer when compared to that of trabeculectomy, with less vision-threatening complications related to hypotony and bleb formation.

### Authors' Comments

From my experience with the iTrack 250A microcatheter (iScience Interventional, CA, USA), it takes about 6 full screw-turns to prime the catheter with Healon G V. You know this is achieved when you see the head of the catheter bending down under the weight of Healon G V. When failing to thread the catheter in one direction (say clockwise), try reversing the direction (counter-clockwise). Turn the screw when retrieving the catheter at 1, 3, 5, 7, 9 and 11 O'clock. Do not stop and wait during retrieval to lessen chances of DM detachment.

### Selective Laser Trabeculoplasty (SLT)

Trabeculoplasty has been in use since the 1970s, but its mechanism has remained uncertain. In recent years, opinion favors that of a cellular and biomechanical cascade, recruiting macrophages in response to trabecular meshwork (TBM) damage, resulting in clearing of debris and improved aqueous outfow. The equilibrium between matrix metalloproteinases (MMP), a family of proteolytic enzymes, and their inhibitors is also altered.^44,45^

Its effects have been well-established, and is useful in OAG, pigment dispersion glaucoma and pseudoexfoliation glaucoma. Its efficacy and safety profle in Asian eyes was found to be favorable in a 5 years study conducted by Lai et al.^46^

In general, SLT achieves a reduction in IOP of 25 to 30%, in around 70% of patients. There is a lack of data on factors predictive of response to SLT. The only factor that seems to correlate with SLT response was a higher baseline IOP. ^47^

Fortunately for POAGLNT patients, there have been studies suggesting SLT to effectively lower mean IOP and IOP variation in POAGLNT patients.^48^ Results by Mallah et al remained statistically significant even when taking confounders such as number of medications and change of medications into account. In addition, as pre-laser use of prostaglandins may lead to a decreased response, this was also looked into, and the distribution of use of prostaglandins and aqueous suppressants remained the same pre and post-laser. One limitation of the study is the limited follow-up period of 9 months. A prospective 3 years study of similar nature conducted in Japan also showed promising results, with a success rate of IOP reduction in 40% at 3 years.^49^

Another potential merit of SLT lies in its repeatability. Hong et al studied the efficacy of repeat 360° SLT in OAG patients with prior successful 360° SLT. Using a definition of success as ≤ 20% reduction from baseline IOP, they found that the success rate of repeat SLT was comparable to that of initial SLT.

Among the abundance of procedures and devices, we need to carefully observe which will stand the test of time. SLT has its disadvantages. We need to explain to patients that there is a lack of data on factors that predict positive response. However, SLT being a noninvasive, quick procedure with a friendly learning curve; not reliant on patient compliance; potentially repeatable with relatively little side effects, remains a very appealing option.

### Authors' Comments

As SLT requires a good view of the trabecular meshwork (TBM), a higher proportion of Chinese patients would require pretreatment with pilocarpine to aid opening up the angle for better visualization. Quite a number of Chinese patients have a narrow angle in the dark without a rise in IOP, this is particularly true if the patient has concomitant cataract. SLT can still be effective if after pilocarpine pretreatment, ≤180° of TBM can be reached partially by the laser spot.

I use alpha-2 agonist as both pre and post-laser medication. Around 15% of patients have IOP spike post-laser. I always check for this 1 hour post-laser, and prescribe oral acetazolamide if present. I do not routinely prescribe steroids or NSAIDs eyedrops, as it has been said that cytokine release plays a role in SLT IOP reduction. I tend to continue all preoperative glaucoma medications and only reduce them carefully after I have judged the IOP lowering effect to be stabilized. I wait at least 1 month before determining the efficacy. I usually treat 360° with 100 shots. I start at 0.8 mJ and titrate down till a champagne bubble reaction is just achieved. In my experience, as low as 0.3 mJ has been noted to be effective. I stay away from areas of peripheral anterior synechiae as I have seen its progression after laser therapy.

### Case Example

Here is an example from our personal experience: A 85 years-old gentleman has a maximum untreated office IOP of 15 mm Hg, vertical cup-to-disk ratio (VCDR) of 0.8, with no systemic hypertension or disk hemorrhage, and CCT was 590 mm. As the PCVI is only 49.7 (the mean PCVI for progressed POAGLNT was 113, that for stable POAGLNT was 69.7), we may like to consider adding glaucoma medications or SLT to achieve a target pressure, as the probability of VF worsening will tend to be low in 3 years' time. Another 60 years old lady who has maximum untreated office IOP of 15 mm Hg, VCDR of 0.8, with systemic hypertension, disk hemorrhage, and CCT of 480 mm, the PCVI would be 227.9. This would represent a more significant risk of progression in coming 3 years, and a surgical option maybe higher on the list especially as her cataract has not been removed yet. Nowadays nonpenetrating surgeries, such as Phaco-NPDS, or phacocanaloplasty, will come earlier in my options offered to patients. Usually the patient will be more acceptable to the surgical options even for the potential complications as the PCVI will serve as a way for the patient to ‘visualize and predict' his or her own future risk of not intervening. For all patients, I shall document their rate of progression (if any), and to readjust the target pressure from time to time. The office IOP fuctuation is another parameter that I would monitor and modulate. The authors appreciate that more studies are needed in the future before we know what is the best clinical decision pathway for patients with POAGLNT.

### Comment on Method Comparison

One reason that makes the comparison of different surgical options that much more difficult is the lack of a standardized method for reporting surgical success and complications in clinical trials. Suggestions to address this have been made by The World Glaucoma Association.^50^ An example for standardizing the report of success suggests that they may include more than one upper limit or a combination of an upper limit and a percentage reduction. Graphical representation of success should clearly illustrate the number of patients in the trial at a particular endpoint. Patients who have achieved a particular endpoint without additional hypotensive medications should be distinguished from those who have required medications. A survival curve plus a scatterplot should be considered a requirement for presentation of trial outcomes data. An example for standardizing the description of complications describes defining bleb leak before initiating the trial and that it should be actively looked for by routinely applying fuorescein stain over a bleb for a positive Seidel's test, rather than doing the test only when hypotony occurs. Bleb leaks should preferably be described as with or without a discrete conjunctival buttonhole, and documentation should be made as to whether an intervention at slit-lamp or inside an operation theater was required.

### CONCLUSION

For POAGLNT patients, before considering surgery, we should always check medication compliance first. If feasible, it would be preferable to have their risk stratifed and progression rate documented, to justify for glaucoma surgery. Balance the risk and benefits incurred from the IOP lowering and IOP modulation. Each surgery option has its inherent advantages and disadvantages. Tailor the management to suit each individual patient to maximize the benefits. When performing drainage surgery, consider combined phacoemulsification to maximize visual, IOP and field benefit.
